# Air pollution and life expectancy: the role of education and health expenditure in China

**DOI:** 10.3389/fpubh.2025.1553039

**Published:** 2025-05-07

**Authors:** Yezhi Zhang, Manshu Huang, Yanshou Zhang, Xuan Kong, Kunpeng Shi, Bingcheng Zhu

**Affiliations:** ^1^School of Design and Fashion, Zhejiang University of Science and Technology, Hangzhou, China; ^2^School of Art, Zhejiang Shuren University, Hangzhou, China; ^3^College of Architecture and Urban Planning, Tongji University, Shanghai, China; ^4^College of Art and Design, Shenyang Ligong University, Shenyang, China; ^5^School of Statistics, Dongbei University of Finance and Economics, Dalian, China

**Keywords:** air pollution, education, health expenditure, life expectancy, China

## Abstract

Health is directly linked to SDG 3, “Good Health and Well-being,” which aims to ensure healthy lives and promote well-being for all ages. Achieving this goal involves improving health outcomes, including increasing life expectancy (LEX) and reducing mortality rates. Therefore, the current study aims to examine the impact of Air pollution’s on LEX, and the role of education, and health expenditure in achieving the SDG 3 in China from 2000 to 2023. This study utilizes the Auto Regressive distributed lag (ARDL) model. We use the Fully modified ordinary least squares (FMOLS) and Dynamic ordinary least squares (FMOLS) methods for robustness analysis. The results show that CO2 emissions and inflation negatively impact LEX, while education, health expenditure, and GDP positively affect LEX. Policymakers should prioritize reducing CO2 emissions through stricter environmental regulations to improve public health. Increased investment in education and healthcare, alongside policies that boost GDP growth, should be emphasized to enhance life expectancy further. Additionally, controlling inflation through sound economic policies will mitigate its negative impact on life expectancy.

## Introduction

Health is a multifaceted concept encompassing social, economic, cultural, and national factors, reflecting social and economic progress and development. Life expectancy (LEX) and mortality rates are commonly used indicators to assess a population’s health conditions and overall health status (1–3). “*LEX refers to the number of years a person can expect to live. By definition, life expectancy is based on an estimate of the average age that members of a particular population group will be when they die*” (4). Another way to define LEX is the number of years that people typically live before dying, not due to an accident or injury, but because people die at a specific age in that group. LEX estimates a nation’s average years of life based on characteristics such as gender differences (5). Many countries regard health care as a fundamental right since it improves people’s quality of life (2, 6). According to Zhang et al. (7) and Coile et al. (8), LEX significantly impacts individual and societal behavior. They pointed out that it has significant implications for intergenerational transfers, economic growth, human capital investment, and incentives for pension benefits (9). Wealthy nations typically spend more on health care and have better health outcomes. However, excessive spending might not always make people live longer (10). LEX is critical for developing nations sincerely seeking socioeconomic advancement through large-scale investments in social sectors such as health, education, sanitation, environmental management and sustainability, and social safety nets (9, 11). Any rise in LEX indicates advances in a society’s human development, well-being, and health. LEX has significantly increased in the globalized world due to developments in healthcare, medicine, and living standards (12, 13). According to Lopreite et al. (14), Changes in demographic structure may lead to a higher prevalence of chronic degenerative diseases, such as heart disease, hypertension, diabetes, cancer, and Alzheimer’s. Over time, this can result in increased disabilities and co-morbidities, which are more costly to manage. Therefore, the demand for long-term care is expected to rise, driving up healthcare expenditures and putting additional strain on the financial sustainability and overall performance of the healthcare system. In Figure A1 shows the Trends of life expectancy at birth rate in China from 1974 to 2023. China’s life expectancy at birth has steadily increased from 60.2 years in 1974 to 78.6 years in 2023. The most rapid growth occurred between the 1970s and 1990s, followed by a more gradual rise in recent decades. Improvements in healthcare, economic development, and living conditions have contributed to this upward trend. Since 2010, the growth rate has slowed, but life expectancy continues to rise.

One of the most potent causes of environmental degradation is considered to be CO2 emissions (CO2E) (15, 16). A population’s overall health and LEX at birth are frequently employed as indicators of LEX (17, 18). Air pollution is an environmental component that contributes to mortality in all societal sectors (19). Rising carbon emissions greatly impact the environment and human health, which lower life expectancy in Europe by increasing respiratory conditions like COPD, asthma, and Rhinosinusitis (20, 21). In developing Asia, life expectancy and public health are adversely affected by carbon emissions, which are made worse by inadequate healthcare systems and the poor health effects of environmental pollution (21). Life expectancy is a key component of the Sustainable Development Goals (SDGs) and a gauge of a country’s health and wellbeing. To increase life expectancy, the United Nations created the Sustainable Development Goals (SDGs), especially SDGs 3, 7, and 13, to reduce environmental pollution and provide everyone with affordable, clean energy (22–24). Reducing industrial changes caused by CO2 will improve public health, lessen inequality, and boost the resilience of people, communities, and society as a whole. According to UNEP research, outdoor environmental pollution costs developing countries about 5% of their GDP (25, 26). Major health hazards associated with air pollution include a significant contribution to premature mortality and the global development of pollution-related disorders (27, 28). According to the World Health Organization, ambient air pollution caused 4.2 million premature deaths globally in 2016, and this figure is expected to rise further as 9 out of 10 people live in areas with poor air quality (29, 30). Environmental degradation may result in unfavorable changes in food production and water quality, which raises death rates, especially for vulnerable individuals from lower socioeconomic backgrounds and baby and older adult populations (29). The populace’s health can be negatively impacted by environmental deterioration in several ways. Increased premature mortality and chronic diseases such as lung cancer, heart disease, and asthma are caused by severe outdoor air pollution (31–33). The longevity of the older adult population is significantly impacted by air quality, since they are less able to handle environmental degradation because of numerous comorbidities (34, 35). Environmental quality greatly impacts human health, which also shapes the values of future generations. Living a long life fosters compassion for future generations, which increases environmental stewardship. Morbidity is increased by pollution, resource degradation, and soil erosion (36, 37). Environmental degradation, especially climate change, can harm population health by changing food production and encouraging heat waves, which raises health risks (38–40).

Education is considered an important predictor of life expectancy. Education has a direct and indirect impact on health outcomes. It increases labor market productivity and economic growth, while educating women improves child health and social wellbeing (11, 41). Degree of education was thought to be a key component in explaining the variations in life expectancy between nations in terms of planning horizon, risk perception in the future, employment of healthcare opportunities, and availability of health-related information (13, 42, 43). Education is often mentioned as one of the main factors influencing lifespan. If everyone had the same death rate as those with tertiary degrees, there would be around 200,000 fewer premature deaths among those aged 30 and over year, according to a strategic review of post-2010 health inequities in England (44, 45). Shorter life expectancy is associated with less education than a high school degree, according to the Reasons for Geographic and Racial Differences in Stroke (REGARDS) study, which included 30,239 Black and White people 45 years of age and older (46). Higher education typically translates into better employment prospects and higher pay, which in turn enable people to enjoy higher living standards and more access to top-notch medical care. By increasing the effectiveness of health production, education can also increase life expectancy (13, 47). Information from the final two to three decades of the 20th century indicates that life expectancy has increased more quickly for persons with greater educational attainment (45, 48). There is a clear correlation between life expectancy and educational attainment across a range of ethnic groups and for both sexes (49, 50). Increased health awareness and a longer life expectancy are the results of education. It also increases women’s production, which benefits the family’s health and the child’s survival. Women with more education had longer life expectancies overall (11, 41). Higher educated people typically lead healthier lifestyles, which lowers the mortality risks related to lifestyle decisions (51). Life expectancy is positively impacted by decreasing fertility rates, which have been associated with education. Significant increases in life expectancy are correlated with higher degrees of education. For example, those who continue their education past the age of 15 are likely to live an additional 14 years (52).

The link between Health expenditure (HE) and health outcomes has been extensively studied in the literature, drawing significant attention from academicians, policymakers, and the general public to the rising healthcare expenditures (53). HE plays a crucial role in improving people’s well-being and supporting a country’s economic development. In both developed and developing nations, there is a strong positive relationship between health spending and economic growth. As economies expand, the demand for medical services rises, leading to greater investments in healthcare. Also, increased life expectancy further drives higher health expenditures, creating a continuous cycle of economic and social development (54). All costs or outlays for medical care, preventive, promotion, rehabilitation, community health initiatives, health administration and regulation, and capital formation with the primary goal of enhancing health are included in the category of health expenditures (55). life span directly rises with increases in life expectancy. Every part of healthcare spending should be enhanced, whether it be through higher-quality human resources or other means (5, 56). Long-term increases in life expectancy and immediate pay increases could result from health spending (57). According to research by Akinci et al. (58), life expectancy rises in the Middle East and North Africa (MENA) when healthcare spending is used efficiently. The nations with the best health results devote significant budgets to medical expenses (59). Spending on health care is better when it is sufficient. On the other hand, low healthcare spending could make it more difficult for the underprivileged to access essential medical treatment (53). Raising health spending lowers baby and under-five death rates and increases life expectancy (60). Countries with lower health expenditures tend to have worse health outcomes. Around 80% of worldwide health spending at the turn of the 20th century was allocated to OECD countries despite making up less than 20% of the world’s population. The poorest three-quarters of the population bore just 7% of health care costs. Despite having 10% of the global population, Africa received only 3% of health spending, whereas Asia and the Pacific, which includes China, received 4% (59, 61–63). Spending on health care by the public and private sectors guarantees access to medical services, and using these services and related goods results in improved health outcomes (64). The effectiveness of public health spending in extending life expectancy starts to plateau when it gets closer to 8% of GDP (65). Enhancing economic development depends heavily on health spending. It raises people’s life expectancy, well-being, and involvement in the productive process (66). A 10% increase in per capita health expenditure is linked to a 3.5-month increase in life expectancy, which suggests that health spending has been the primary driver of longevity advances in recent decades, according to the OECD (67, 68). Longevity has been observed to increase with increases in healthcare spending and the extension of healthcare services, particularly for the older adult (68, 69). Short-term health-related facilities, such as the availability of doctors and hospital beds, impact life expectancy. On the other hand, persistent investments in health technology and infrastructure might be necessary for long-term gains (70).

China was selected as the study sample because, in 2023, it emitted 11.9 billion metric tons of carbon dioxide, making it the world’s largest emitter that year. While many countries saw significant reductions in emissions in 2020 due to the COVID-19 pandemic, China was one of the few nations where emissions increased during that period. China has ambitious carbon neutralization goals for 2060, marking a pivotal moment in the global energy transition. This transition is driven by China’s commitment to ecological civilization, technological advancements, progressive environmental policies, and significant changes in energy production and consumption systems. Over recent years, China has made substantial investments in the production of green energy, which now accounts for 50% of the country’s total energy output. Additionally, China has expanded healthcare coverage with initiatives such as the New Cooperative Medical Scheme (NCMS) in rural areas and the Urban Resident Basic Medical Insurance (URBMI) in cities. The “Healthy China 2030” plan, introduced in October 2016, aims to improve national health by ensuring lifelong wellness for all citizens, integrating health considerations into all policy areas, and striving to increase life expectancy to 79 years by 2030. The “Double First-Class” Construction Policy is a strategic educational initiative in China to develop world-class universities and disciplines. In 2015, the State Council of China introduced the “Overall Plan for Coordinated Promotion of the Initiative of World-Class Universities and First-Class Disciplines,” emphasizing enhancing China’s higher education system. This policy targets universities that already possess high-level disciplines and play a leading role in China’s education sector. It encourages them to establish additional first-class disciplines around their core strengths, refine their academic focus, and expand their global influence. The ultimate goal is to position these institutions at the forefront of the international education system, fostering excellence and innovation in higher education (71). In October 2016, President Xi Jinping introduced the Healthy China (HC) 2030 blueprint, a landmark initiative that prioritizes public health as a fundamental prerequisite for future economic and social development. As a national strategy, HC 2030 aims to promote universal participation in health, shared health benefits, and individual responsibility for well-being. It establishes five specific goals: enhancing overall health levels, controlling major risk factors, strengthening healthcare service capacity, expanding the health industry, and improving the healthcare system. The blueprint is guided by four core principles: health priority, reform and innovation, scientific development, and justice and equity. Additionally, it defines 13 key indicators to track progress, with evaluation milestones set for 2020 and 2030 to measure the effectiveness of health policies and reforms (72). The HC 2030 blueprint outlines five key targets: improving overall health levels, controlling major risk factors, enhancing healthcare service capacity, expanding the health industry, and strengthening the healthcare system. By 2030, HC 2030 aims to significantly enhance public health, raising the average life expectancy to 79 years, increasing health literacy, and promoting healthy lifestyles. It also emphasizes the development of energy-efficient and environmentally friendly industries, ensuring food and drug safety and reducing the risk of severe illnesses. Moreover, the plan focuses on building an integrated, high-quality healthcare system, refining public health security, advancing health science and technology, and improving the quality and accessibility of health services. HC 2030 also seeks to establish a modernized health industry, fostering innovative and globally competitive enterprises, strengthening health-related laws and regulations, and achieving modern governance in the health sector (72). The Double First-Class policy enhances China’s global academic standing by developing world-class universities and disciplines, fostering innovation, and strengthening international collaboration. The Healthy China 2030 initiative improves public health by promoting disease prevention, expanding healthcare services, and ensuring a sustainable and high-quality medical system. Both policies contribute to China’s long-term social and economic development by prioritizing education and health as key drivers of national progress.

This study contributes to the existing literature in several ways. Firstly, it examines the combined impact of air pollution, education (EDU), and health expenditure on LEX in China from 2000 to 2023, a broader approach compared to previous studies that focused on individual factors. Secondly, it incorporates GDP and inflation as important variables affecting LEX, adding to the comprehensiveness of the analysis. Thirdly, the study offers valuable insights for policymakers, providing a more nuanced understanding of the factors influencing life expectancy in China. Lastly, the study employs a robust methodology, enhancing the reliability and relevance of its findings. The rest of the study is organized as follows: Section 2 of the literature review sheds light on the previous studies. Section 3 covers the model, methodology, and data. Section 4 illustrates the findings and discussion. Section 5 covers the conclusion and policy recommendation.

## Literature review

Bilas et al. (73) examined the factors influencing LEX at birth in the 28 European Union countries from 2001 to 2011 using panel data and linear regression. They found that higher education levels were associated with a decrease in LEX, contrary to common belief. Specifically, a 1% increase in education correlated with a 0.055% drop in LEX, assuming GDP per capita remained constant. Duba et al. (74) analyzed the impact of healthcare expenditures on LEX in 210 countries and regions from 1995 to 2014, using a fixed-effects regression model. They found a positive correlation between education and LEX, suggesting that individuals with higher education tend to lead healthier lifestyles. Jafrin et al. (75) studied the determinants of LEX in five SAARC countries from 2000 to 2016 using panel data estimation. They found that longer schooling years were positively linked to higher LEX, suggesting that increased educational investment could promote healthier lifestyles and better health outcomes. Sede and Ohemeng (9) used the Vector Auto-Regression method to analyze LEX in Nigeria from 1980 to 2011. They found that traditional socioeconomic factors such as per capita income, education, and government health spending had limited influence on LEX in Nigeria. Azam et al. (22) explored the determinants of LEX in Pakistan from 1975 to 2020 using the ARDL technique. They found that education positively impacted LEX in the short and long term, with a 1% increase in education leading to a 0.02002% rise in LEX. Abubakari et al. (76) analyzed LEX in 44 Sub-Saharan African countries from 2000 to 2015 using the Generalized Method of Moments. They found that LEX was positively impacted by GDP per capita, health spending, and education, particularly secondary school enrollment. Bayati et al. (77) studied LEX in 21 Eastern Mediterranean countries from 1995 to 2007 using panel data and fixed-effects models. They found that education strongly influenced LEX, with individuals who had higher education being more proactive about their health. Kabir (11) examined LEX in 91 developing countries using multiple regression and probit models. He found that adult illiteracy had a detrimental effect on LEX. Gilligan and Skrepnek (78) studied LEX in the Eastern Mediterranean Region from 1995 to 2010, using a multilevel mixed-effects linear model. They found that adult literacy was a significant predictor of LEX. Moga Rogoz et al. (13) analyzed the impact of economic freedom and education on LEX in new EU member states from 2000 to 2019. They found that economic freedom and education significantly impacted LEX, with education having a more substantial effect. Delavari et al. (79) investigated the relationship between LEX and its socioeconomic determinants in Iran from 1985 to 2013 using an ordinary least-square model. They found a strong positive link between LEX and literacy rates. Using the ARDL, Şentürk and Ali (80) analyzed gender-specific LEX in Turkey from 1971 to 2017. They found that LEX was influenced by economic development, educational attainment, and environmental factors, with female education significantly impacting LEX for women. Hassan et al. (81) studied LEX in 108 developing countries from 2006 to 2010 using a panel data technique and the Vector Error Correction Model. They found a positive relationship between LEX and education, GDP, health expenditure, and sanitation facilities. Novak et al. (82) explored the role of education in LEX in 187 countries from 2005 to 2010. They found that education was a significant predictor of LEX, with a positive relationship between educational attainment and LEX. Zhang et al. (83) analyzed the impact of education, urbanization, and green growth on LEX in china from 1990 to 2022 by using the ARDL technique, they found that education, urbanization, and green growth have positive effect on LEX in china.

Morina et al. (84) investigated the effect of health expenditure on LEX from 2005–2018. They applied random and fixed effect models. The study found that health expenditures positively impact LEX in OECD countries, demonstrating the importance of economic development and careful management of public health spending to improve health outcomes and longevity. Duba et al. (74) explored the effects of healthcare expenditures on LEX in 210 countries from 1995 to 2014, using a fixed effects regression model. The findings showed a statistically significant positive correlation between healthcare spending and LEX, especially in poorer countries. Deshpande et al. (85) analyzed the relationship between national healthcare expenditure and LEX in 181 countries. Their findings indicated no significant association between healthcare spending and LEX in low-income countries but a positive relationship in wealthier nations. Behera and Dash (86) studied the effect of health expenditure on achieving healthcare goals in 10 Southeast Asian countries from 2000 to 2014, using a system GMM. They found that aggregate and public health expenditures positively influenced LEX and reduced infant mortality. Rhee (70) examined the effects of healthcare expenditure on infant mortality and LEX in Korea from 1985 to 2010. Using Ordinary Least Squares regression and Granger causality tests, they found a positive connection between healthcare expenditure and LEX. Rezapour et al. (2) explored the impact of health expenditure on health outcomes in high-income countries from 2000 to 2015 using panel data regression models. They found that public health spending significantly influenced LEX and reduced infant and under-five mortality. Sabra (66) studied the nexus between health expenditure and LEX in six middle-income MENA countries from 2000 to 2019. The results showed a strong positive relationship between health expenditure and LEX, recommending increased health spending and education investments to improve human development and LEX. Using a two-phase statistical model, Deluna and Peralta (55) examined the relationship between public health expenditures, income, and health outcomes in the Philippines from 1981 to 2010. They found that health expenditure positively impacted LEX, suggesting sufficient healthcare spending is essential for improving LEX and reducing child mortality. Using dynamic panel data models, Ray and Linden (62) analyzed the relationship between health expenditure, LEX, and child mortality in 195 countries from 1995 to 2014. They found that public health expenditures had a more significant impact on health outcomes than private expenditures.

Liu and Zhong (53) examined the relationship between health spending, LEX, and renewable energy in China from 2000 to 2020 using the VECM technique. They found that increased health spending had a long-term positive effect on LEX. Zhang (87) studied the impact of green taxes and public health spending on LEX in China using GMM regression models. The findings showed that both green taxes and public health spending had a significant positive effect on LEX. Khan et al. (25) investigated the influence of government effectiveness, health expenditure, and sustainable development goals (SDGs) on LEX in Pakistan from 2000 to 2020, using Johansen Cointegration test. They found that health expenditure and SDGs significantly impacted LEX, while government effectiveness had a negative relationship. Using pooled regression and pairwise correlation methods, Sango-Coker and Bein (5) analyzed the impact of healthcare spending on LEX in selected West African countries from 1999 to 2014. They found a positive association between public sector healthcare expenditure and LEX. Boundioa and Thiombiano (88) examined the threshold influence of governance quality on the relationship between public health expenditure and LEX in the West African Economic and Monetary Union from 1996 to 2018. They found that governance quality is a transition variable influencing the effect of public health spending on LEX.

Mahalik et al. (28) investigated how CO2E affected LEX in 68 low- and middle-income countries from 1990 to 2017. Using panel econometrics, they found negative and positive effects for emerging economies for developing countries. The study highlighted that consumption and production-based emissions reduce LEX in emerging economies. Murthy et al. (89) examined the relationship between CO2E, economic growth, and LEX in D-8 countries from 1992 to 2017 using panel ARDL methods; they found that CO2E significantly negatively affected LEX. Economic and population growth and health expenditure were found to influence LEX positively. Osabohien et al. (90) aimed to explore how energy consumption and CO2E impacted LEX in Nigeria from 1980 to 2017 by using the ARDL model. The results showed a significant negative impact of carbon emissions on LEX. Azam and Adeleye (91) investigated the impact of liquid and solid fuel emissions on LEX in 36 Asia-Pacific countries between 2005 and 2010. They employed GMM method. The study found significant negative effects from both liquid and solid fuel emissions. Liquid fuel emissions had the most adverse impact on LEX. Das and Debanth (17) aimed to analyze the relationship between CO2E and LEX in India from 1991 to 2018. The study used the ARDL cointegration technique. The results indicated that India has surpassed its optimal CO2E concentration, negatively affecting LEX. Amuka et al. (92) examined the effect of CO2E on LEX in Nigeria from 1995 to 2013 by using OLS regression; they found a positive but statistically insignificant relationship between CO2E and LEX. Redzwan and Ramli (23) analyzed the relationship between CO2E, GDP, health expenditure, and LEX in Malaysia from 1997 to 2021. The ARDL bounds test revealed significant relationships between carbon emissions and health expenditure with LEX. Uddin et al. (93) investigated the role of institutional quality, financial development, and environmental degradation (proxied by CO2E) in determining LEX across selected Asian countries from 2002 to 2020. Using CS-ARDL, FMOLS, and DOLS methods, they found that CO2E and ecological footprint reduce LEX. Chen and Li (94) examined the impact of CO2E on LEX in china by using the Computable General Equilibrium (CGE). The results of the CGE model indicate that GDP contributes to an increase in LEX, while emissions have a negative effect, shortening LEX.

Building on the existing literature, we identify the following gap: Previous studies have individually examined the effects of air pollution, education, and health expenditure on LEX in China. For instance, Zhang et al. (83) explored the relationship between education and LEX, while Liu and Zhong (53) and Zhang (87) focused on health expenditure and its impact on LEX. Similarly, Chen and Li (94) analyzed the effect of CO2 emissions on LEX. However, none of these studies have considered the combined impact of these factors on life expectancy in the context of the Chinese economy. Moreover, inflation has been overlooked in prior research, creating another gap. This study addresses both gaps by analyzing the joint effect of air pollution, education, health expenditure, and inflation on life expectancy in China, offering a more comprehensive understanding of the factors influencing public health outcomes.

## Methodology and data

### Empirical model

The main purpose of this study to analyze the impact of Air pollution, Education and health expenditure on life expectancy in china. This following model use in this study emerged from the previous literature.


(1) $$\begin{array}{l} {LEX}_{t}=\omega_{0}+\omega_{1}CO2E_{t}+\omega_{2}{EDU}_{t}+\omega_{3}{\mathrm{HE}}_{t}+\\ \omega_{4}{INF}_{t}+\omega_{5}{GDP}_{t}+e_{t} \end{array}$$

In Equation 1, LEX, CO2E, EDU, HE, INF and GDP signifies the life expectancy, CO2 emissions, education, health expenditure, inflation and gross domestic product, respectively. CO2E used as a proxy for Air pollution as also used by Muradov et al. (95) and Ebhota et al. (96). Air pollution is a major environmental threat to human health, regardless of a country’s income level. According to the World Health Organization (WHO), 99% of the global population is exposed to air pollution levels exceeding guideline limits. This exposure contributes to increased premature mortality, a rise in chronic diseases, and reduced LEX. Recent studies indicate that prolonged exposure to air pollution significantly raises the risk of cardiovascular and respiratory diseases, shortening LEX by an average of 1 to 8 years worldwide (95).

It is expected that $\omega_{2}>0$ , showing that education has positive effect on LEX. Education enhances LEX by fostering healthier lifestyles, improving access to healthcare, and increasing health literacy. Educated individuals are more aware of health risks, more likely to adopt preventive measures, and make informed choices regarding diet, exercise, and medical care. Additionally, education improves economic opportunities, reducing poverty-related health risks while ensuring better access to clean water, sanitation, and nutritious food (83).

It is expected that $\omega_{3}>0$ , it shows that health expenditure has positive effect on LEX, In recent decades, healthcare expenditures have become a key measure of government commitment to public well-being. A rise in healthcare spending as a share of GDP is associated with increased life expectancy at birth, highlighting the significant benefits of investing in public health. This relationship underscores the importance of adequate funding in improving overall population health (97). The coefficient of inflation is expected to negative effect on LEX, Inflation is typically accompanied by a rise in the overall price level, reducing the purchasing power of money. As a result, it affects health outcomes by increasing healthcare costs. High inflation drives up the prices of medical services, medications, and other health-related necessities, making them less accessible for many individuals. This financial burden limits access to proper healthcare, leading to greater reliance on unqualified practitioners, poorer living conditions, inadequate nutrition, and ultimately, higher mortality rates (22). It is also expected that GDP has positive effect on LEX, according to Țarcă et al. (98) Economic growth and development, reflected in GDP per capita, contribute to longer life expectancy by improving living standards, healthcare access, and overall well-being. As a country’s economy expands, its citizens generally experience increased longevity and a higher quality of life. The term $\omega_{0}$ and $\omega_{1}\;to\;\omega_{5}$ shows the intercept and slope coefficients of the CO2E, EDU, HE, INF and GDP variables. LEX is dependent variable and CO2E, EDU, HE, INF and GDP are explanatory variables. All variables are converted to natural logarithms except INF and HE.

### Methodology

In this study, we initially employ unit root tests such as the Augmented Dickey-Fuller (ADF), Phillips-Perron (PP) and Kwiatkowski, Phillips, Schmidt, and Shin (KPSS) tests to examine the stationarity of the data. The ADF test, introduced by Dickey and Fuller (99), is based on Equation 2:


(2) $$\Delta G_{t}=\omega ++\emptyset t+\partial G_{t-1}+\overset{p}{\underset{i=1}{\sum }}\delta_{i}\Delta G_{t-i}+u_{t}$$

Where in Equation 2, $\Delta G_{t}$ , ω , ∅t , $\partial G_{t-1}$ , $\overset{p}{\underset{i=1}{\sum }}\delta_{i}\Delta G_{t-i}$ and $u_{t}$ represent the first difference of the dependent variable, drift term, deterministic trend, lagged level of the series, lagged differences of the dependent variable, and white noise error term, respectively. The null hypothesis (H0): ∂=0 indicates the presence of a unit root, while the alternative hypothesis (H1): ∂<0 suggests stationarity (100).

Secondly, we employed the Johansen Cointegration test, developed by Johansen (101), is used to determine the number of Cointegration relationships between multiple time series. It relies on maximum likelihood estimation to test for the presence of Cointegration and identify the number of Cointegrating vectors. The test includes two procedures: the trace test and the maximum eigenvalue test. After the Cointegration we employs the Autoregressive Distributed Lag (ARDL) model, developed by Pesaran et al. (102). The ARDL model is mainly helpful for estimating both short-run and long-run relationships between variables, especially when the variables exhibit a mixed order of integration, i.e., when some are I(0) (stationary at level), and others are I(1) (stationary after first differencing). ECM, derived from a Cointegration relationship, focuses on short-run adjustments towards long-run equilibrium. ARDL uses the bounds test for Cointegration, while ECM typically relies on Johansen’s test. A key benefit of ARDL is its strong performance in small samples, making it more practical for limited data. The ARDL model is specified as in Equation 3:


(3) $$\begin{array}{l} \Delta LEX_{t}=\phi_{0}+\omega_{1}LEX_{t-1}+\omega_{2}CO2E_{t-1}+\omega_{3}EDU_{t-1}+\\ \omega_{4}HE_{t-1}+\omega_{5}\mathrm{IN}F_{t-1}+\omega_{6}GDP_{t-1}+\\ \phi_{5}e_{t-1}+\overset{n}{\underset{i=1}{\sum }}\gamma_{1}\Delta LEX_{t-1}+\overset{n}{\underset{i=0}{\sum }}\gamma_{2}\Delta CO2E_{t-1}+\\ \overset{n}{\underset{i=0}{\sum }}\gamma_{3}\Delta EDU_{t-1}+\overset{n}{\underset{i=0}{\sum }}\gamma_{4}\Delta HE_{t-1}+\\ \overset{n}{\underset{i=0}{\sum }}\gamma_{5}\Delta INF_{t-1}+\overset{n}{\underset{i=0}{\sum }}\gamma_{6}\Delta GDP_{t-1}+e_{t} \end{array}$$

The term Δ difference operator. The null hypothesis, which states that no long-run relationship exists between the variables ( $H_{0}:\omega_{1}=\omega_{2}=\omega_{3}=\omega_{4}=\omega_{5}==\omega_{6}=0$ ) is tested using the F-statistic. If the F-value is less than the lower bound, H0 is accepted, indicating no Cointegration among the variables. Conversely, if the F-value exceeds the upper bound, Ho is rejected, confirming the presence of Cointegration. However, if the F-value falls between the lower and upper bounds, the result is inconclusive. To estimate short-run relationships, the Error Correction Model (ECM) is specified in Equation 4:


(4) $$\begin{array}{l} \Delta LEX_{t}=\phi_{0}+\overset{n}{\underset{i=1}{\sum }}\gamma_{1}\Delta LEX_{t-1}+\overset{n}{\underset{i=0}{\sum }}\gamma_{2}\Delta CO2E_{t-1}+\\ \overset{n}{\underset{i=0}{\sum }}\gamma_{3}\Delta EDU_{t-1}+\overset{n}{\underset{i=0}{\sum }}\gamma_{4}\Delta HE_{t-1}+\\ \overset{n}{\underset{i=0}{\sum }}\gamma_{5}\Delta INF_{t-1}+\overset{n}{\underset{i=0}{\sum }}\gamma_{6}\Delta GDP_{t-1}+\\ \varphi_{1}ECM_{t-1}+e_{t} \end{array}$$

A negative and statistically significant $ECM_{t-1}$ coefficient $\left(\varphi_{1}\right)$ indicates that any short-term disequilibrium between the dependent and explanatory variables will adjust and converge toward the long-run equilibrium relationship (83). For the robustness analysis we employed the FMOLS and DOLS estimators. The FMOLS (Fully Modified Ordinary Least Squares), developed by Phillips and Hansen (103), is designed to estimate long-run relationships in cointegrated systems by addressing serial correlation and endogeneity through non-parametric adjustments to the residuals. This method ensures asymptotically valid t-statistics and is particularly effective in small sample sizes. DOLS (Dynamic Ordinary Least Squares), introduced by Stock and Watson (104), extends the OLS method by incorporating leads and lags of the first differences of explanatory variables to correct for endogeneity and serial correlation. Both methods produce unbiased and efficient parameter estimates for cointegrated relationships, with FMOLS offering non-parametric corrections and DOLS leveraging dynamic interactions for robustness and simplicity in applied econometric studies (83).

### Data and variables

This study examines the impact of CO2E, EDU and HE on LEX in china. The data has been obtained from world development indicator (WDI) and United Nations Development Programme (UNDP) from 2000 to 2023. Table 1 shows the data sources.

**Table 1:** Variable measurement.

Symbols	Variables	Measurement	Sources
LEX	Life expectancy	Birth, total (years)	WDI
CO2E	Carbon emissions	Metric tons per capita	
EDU	Education	Mean years of schooling	UNDP
HE	health expenditure	% of GDP	WDI
INF	Inflation	Annual %	
GDP	Gross domestic product	GDP per capita (constant 2015 US$)	

## Results and discussions

### Descriptive statistics

Table 2 provides the descriptive statistics for the study variables. The mean values of life expectancy, CO2 emissions, education, health expenditure, inflation, and gross domestic product are 4.328, 9.036, 13.061, 4.653, 2.093, and 8.660, respectively. The standard deviations for these variables are 0.026, 0.400, 1.711, 0.533, 1.708, and 0.549, respectively. Lastly, the Jarque-Bera statistics confirm that all variables are normally distributed, with highly insignificant results, supporting the null hypothesis of normality.

**Table 2:** Descriptive statistics.

	LEX	CO2E	EDU	HE	INF	GDP
Mean	4.328	9.036	13.061	4.653	2.093	8.660
Median	4.331	9.223	13.221	4.537	1.948	8.759
Maximum	4.364	9.451	15.218	5.594	5.925	9.407
Minimum	4.275	8.205	9.905	3.675	−0.732	7.693
Std. Dev.	0.026	0.400	1.711	0.533	1.708	0.549
Skewness	−0.373	−0.881	−0.356	0.104	0.563	−0.340
Kurtosis	2.010	2.453	1.936	1.962	3.091	1.811
Jarque-Bera	1.536	3.403	1.640	1.121	1.275	1.875
Probability	0.464	0.182	0.440	0.571	0.529	0.392

### Unit root and Cointegration test

Table 3 presents the outcomes of the ADF, PP and KPSS unit root test. The outcomes of ADF reported that with constant LEX, CO2E, INF and GDP are stationary at level and EDU and HE are Non Stationary at level. INF is stationary at level With Constant & Trend and LEX, CO2E, EDU, HE and GDP are non-stationary at level. After the first difference all variables becomes stationary. The results of PP reported that with constant all variables are stationary at level, while With Constant & Trend only INF is stationary. At level the KPSS test shows that with constant and With Constant & Trend all variables are stationary at level except HE. After the first difference all variables becomes stationary. Table 4 presents the results of the Johansen Cointegration test and the ARDL bound test. The Johansen Cointegration test, based on the Trace statistics, identifies 5 cointegrated Cointegration equations, while the Max-Eigen statistics also confirms 5 Cointegration equations. Additionally, Table 4 displays the results of the ARDL bound test, where the F-statistic (5.61) is greater than the critical value (4.15), indicating that there is Cointegration among the variables.

**Table 3:** Unit root test.

ADF							
At level							
		LEX	CO2E	EDU	HE	INF	GDP
With constant	t-statistic	−3.994^*^	−4.841^*^	−2.553	−0.256	−4.000^*^	−4.437^*^
With constant & trend		−0.674	−0.603	−0.693	−1.993	−3.492^**^	0.614
At first difference							
		∆(LEX)	∆(CO2E)	∆(EDU)	∆(HE)	∆(INF)	∆(GDP)
With constant	t-statistic	−4.078^*^	−1.345	−2.311	−4.037^*^	−7.839^*^	−0.731
With constant & trend		−5.075^*^	−3.309^**^	−3.573^**^	−3.950^*^	−8.701^*^	−4.323^*^

PP							
At level							
		LEX	CO2E	EDU	HE	INF	GDP
With constant	t-statistic	−5.793^*^	−4.741^*^	−3.547^**^	−0.374	−3.938^*^	−3.801^*^
With constant & trend		−1.6	−0.603	0.13	−2.348	−3.732^**^	0.445
At first difference							
		∆(LEX)	∆(CO2E)	∆(EDU)	∆(HE)	∆(INF)	∆(GDP)
With constant	t-statistic	−4.025^*^	−1.345	−1.408	−4.026^*^	−8.758^*^	−2.032
With constant & trend		−9.630^*^	−3.309^**^	−3.694^**^	−3.918^*^	−11.063^*^	−4.348^*^

KPSS							
At Level							
		LEX	CO2E	EDU	HE	INF	GDP
With constant	LM-stat.	0.225^a^	0.131^a^	0.302^a^	0.558	0.169^a^	0.699
With constant & trend		0.195^a^	0.185^a^	0.176^a^	0.441	0.170^a^	0.186^a^
At first difference							
		∆(LEX)	∆(CO2E)	∆(EDU)	∆(HE)	∆(INF)	∆(GDP)
With constant	LM-stat.	0.120^a^	0.120^a^	0.012^a^	0.232^a^	0.291^a^	0.313^a^
With constant & trend		0.132^a^	0.098^a^	0.079^a^	0.128^a^	0.176^a^	0.119^a^

Note: *, ** & *** indicates the significance level at 1%, 5% & 10%. KPSS critical values at 1%, 5% & 10% are 0.739, 0.463 & 0.347 respectively and superscript a represent the acceptance of (Ho) of KPSS. Ho: of KPSS is stationary.

**Table 4:** Cointegration test.

Hypothesized	Trace			Max-eigen	
No. of CE(s)	Eigenvalue	Statistic	Prob.	Statistic	Prob.
None	0.993	255.745^*^	0.000	109.374^*^	0.000
At most 1	0.900	146.371^*^	0.000	50.728^*^	0.000
At most 2	0.825	95.643^*^	0.000	38.337^*^	0.001
At most 3	0.660	57.307^*^	0.000	23.709^*^	0.021
At most 4	0.649	33.597^*^	0.000	23.005^*^	0.002
At most 5	0.382	10.592^*^	0.001	10.592^*^	0.001

Test statistic	Value	Critical value	Test statistic	Value
F-statistic k	5.61^*^ 5	Significance	I (0)	I (1)
		10% 5% 1%	2.08 2.39 3.06	3.0 3.38 4.15

*, ** & *** indicates the significance level at 1, 5% & 10%.

### ARDL estimation results

Table 5 presents the ARDL test results. The long-run coefficient of CO2E is −0.339, suggesting that a 1% increase in CO2E leads to a 0.339% decline in life expectancy (LEX). This negative impact arises because CO2E contributes to air pollution, which increases the risk of respiratory and cardiovascular diseases, ultimately reducing LEX. Prolonged exposure to high CO2E and other pollutants deteriorates public health, increases mortality rates, and shortens lifespans. Climate change driven by CO₂ emissions also exacerbates extreme weather events, food insecurity, and the spread of infectious diseases, further negatively affecting life expectancy. These findings are consistent with Azam et al. (22) and Uddin et al. (93), who reported similar adverse effects of CO2E on LEX in Pakistan and South Asia.

**Table 5:** ARDL estimates.

	Long run			Short run		
Variable	Coefficient	Std. Error	Prob.	Coefficient	Std. Error	Prob.
CO2E	−0.339^**^	0.125	0.014	−0.023^**^	0.008	0.017
EDU	0.217^*^	0.024	0.000	0.006^***^	0.003	0.085
HE	0.071^***^	0.039	0.084	0.005^**^	0.002	0.025
INF	−0.197^*^	0.058	0.001	−0.028^***^	0.014	0.065
GDP	0.434^**^	0.178	0.025	0.050^*^	0.013	0.003
ECM(−1)				−0.826^*^	0.000	0.000

ARDL diagnostic test				
Test	F-stats	*p*-value		
LM test for Autocorrelation	1.045	0.243		
Ramsey RESET test for stability	0.136	0.719		
ARCH test for Heteroscedasticity	0.687	0.417		
Jarque–Bera test for normality	0.237	0.888		

*, ** & *** indicates the significance level at 1, 5% & 10%. Selected model: ARDL (1, 1, 1, 1, 1). Dependent variable LEX.

The coefficient for EDU is 0.217, indicating that a 1% increase in EDU raises LEX by 0.217%, with statistical significance at the 1% level. Education enhances life expectancy by promoting healthier behaviors, increasing access to healthcare, and improving health literacy. People with higher education are more likely to understand health risks, adopt preventive healthcare measures, and make informed diet, exercise, and medical care decisions. Furthermore, education creates economic opportunities, reducing poverty-related health risks and improving access to clean water, sanitation, and nutritious food. These results align with Liu et al. (105), Iyakaremye and Tripathi (106), and Bijwaard et al. (107). Liu et al. (105) found that individuals with higher education tend to report better health, maintain a healthier weight, and engage less in harmful behaviors such as smoking or excessive alcohol consumption. Iyakaremye and Tripathi (106) highlighted that higher education is linked to lower fertility rates and increased life expectancy, particularly among those with advanced education levels. Zhang et al. (83) found that education has positively affect LEX in china. Bijwaard et al. (107) found that education significantly impacts life expectancy, with individuals with higher education generally living longer, noting that knowledge provides additional advantages for survival.

The coefficient of HE is positive, showing that a 1% increase in health expenditure leads to a surge in LEX by 0.071%. Health expenditure positively impacts LEX by improving access to quality healthcare, medical treatments, and preventive services. Increased spending enables better healthcare infrastructure, reducing mortality rates and enhancing disease management. It also supports public health initiatives, improving overall population health. As a result, higher health expenditure leads to longer life expectancy by addressing both immediate health needs and long-term health outcomes. The findings are consistent with those of Morina et al. (84), Duba et al. (74), Deshpande et al. (85), and Behera and Dash (86). Morina et al. (84) demonstrated that health expenditures positively influence life expectancy in OECD countries, emphasizing the importance of efficient public health spending to improve longevity. Duba et al. (74) found a significant positive correlation between healthcare spending as a percentage of GDP and life expectancy in 210 countries, suggesting that increasing healthcare investment, especially in low-income countries, could enhance public health outcomes. Deshpande et al. (85) observed that healthcare expenditure had no significant effect on life expectancy in low-income countries, but a notable impact in developed nations. Behera and Dash (86) found that both aggregate and public health expenditures positively affect life expectancy, recommending increased public spending on health to improve health outcomes and reduce infant mortality.

The Chinese government made a wonderful Initiative for education and health, implemented the Double First-Class Initiative, improved literacy, reduced poverty, and fostered social mobility. Public health policies, like the New Rural Cooperative Medical Scheme and Healthy China 2030 Plan, expanded healthcare access and reduced mortality rates. These reforms enhanced health awareness, economic stability, and equitable development. The synergy between education and healthcare created a healthier, more educated population, driving China’s economic growth and improving overall quality of life. These efforts have significantly increased LEX and reduced disparities across urban and rural areas.

The coefficient of INF is negative, showing that a 1% inflation increase leads to a LEX reduction by 0.197%. The reason for the negative sign is that Inflation negatively impacts life expectancy by reducing the affordability of healthcare and essential goods, such as nutritious food and medicines, especially for low-income households. As inflation rises, people may prioritize basic needs over health, leading to poorer health outcomes and higher mortality rates. Due to rising costs, strained healthcare systems can result in limited treatment access and worsening public health. Increases in inflation may also lead to higher poverty levels, further diminishing life expectancy in vulnerable populations. The coefficient of GDP is positive, showing that a 1% increase in inflation leads to a surge in LEX by 0.434%. The reason for the positive is that GDP positively impacts life expectancy by enabling governments to invest in healthcare infrastructure, education, and social services. Higher GDP often leads to better access to medical care, improved nutrition, and healthier living conditions. Economic growth can reduce poverty, which is closely linked to better health outcomes and longer life expectancy. Additionally, higher GDP can promote technological advancements and innovations in healthcare, further extending life expectancy. The finding is consistent with the line of Uddin et al. (93) and Azam et al. (22).

Table 5 presents the short-run ARDL estimates for China from 2000 to 2023, showing that EDU, HE, INF, and GDP positively affect LEX, while INF and CO2E negatively impact LEF. The error correction mechanism (ECM) coefficient is −0.826 and statistically significant. This negative coefficient implies that 82.6% of the deviation in the dependent variable is corrected and returns to equilibrium in each period. These results suggest a rapid adjustment towards long-run equilibrium following short-term shocks.

### ARDL diagnostic and robustness analysis

**Figure 1:**
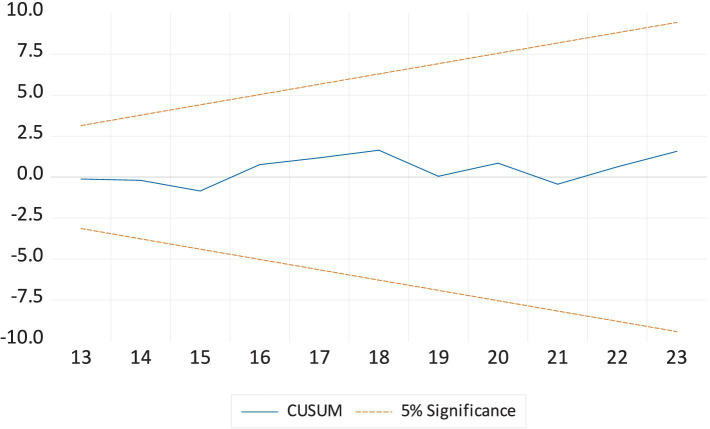
The cumulative sum of the recursive residual plot.

**Figure 2:**
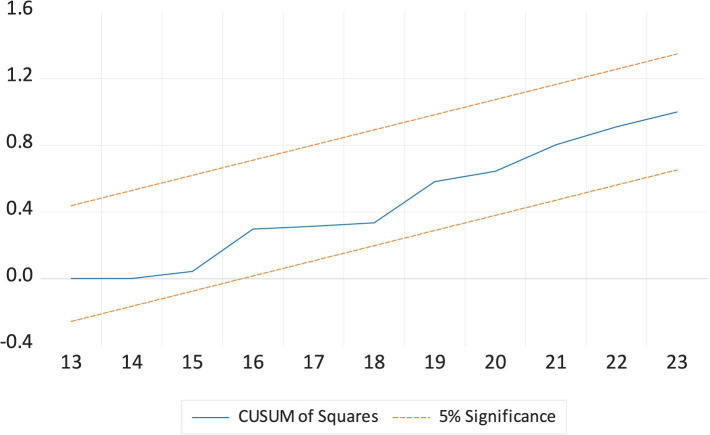
The cumulative sum of the square of the recursive residual plot.

Table 6 presents the ARDL diagnostic analyses for the LEX models. The diagnostic tests indicate that autocorrelation and heteroscedasticity do not pose issues in the ARDL model. Both the Ramsey RESET test and the Jarque-Bera statistic accept the null hypothesis of model stability and normal distribution. Figures 1, 2 further confirm that the model parameters are free from errors. These results suggest that the model provides reliable and accurate outcomes. Table 6 shows the robustness analysis using FMOLS and DOLS confirms the ARDL long-run estimates, with some variation in magnitude and significance levels. The results indicate that EDU, HE, and GDP positively affect LEX, while INF and CO2E negatively impact LEX.

**Table 6:** Robustness analysis.

	FMOLS			DOLS		
Variable	Coefficient	Std. error	Prob.	Coefficient	Std. error	Prob.
CO2E	−0.291^**^	0.111	0.043	−0.776^*^	0.186	0.000
EDU	0.216^*^	0.034	0.000	0.385^*^	0.043	0.000
HE	0.582^*^	0.054	0.000	0.265^**^	0.115	0.050
INF	−0.706	0.010	0.000	−0.720^*^	0.093	0.000
GDP	0.490^***^	0.244	0.060	0.576	0.315	0.319

*, ** & *** indicates the significance level at 1, 5% & 10%.

Table 7 shows the Granger causality test estimates. The Granger causality results indicate a unidirectional relationship from CO2E to LEX at the 1% significance level, suggesting that CO2E significantly influences LEX but not vice versa. EDU, HE, INF, and GDP also Granger-cause LEX at varying significance levels, implying their predictive power over life expectancy. However, LEX does not Granger-cause any of these variables, indicating that changes in life expectancy do not significantly predict them. Additionally, HE Granger-causes CO2E, and CO2E, in turn, Granger-causes HE, suggesting a bidirectional relationship. Lastly, CO2E also Granger-causes GDP, while GDP does not significantly predict CO2E, indicating a one-way effect.

**Table 7:** Granger causality.

Null hypothesis	F-statistic	Prob.
CO2E does not granger cause LEX	11.661^*^	0.001
LEX does not granger cause CO2E	0.620	0.550
EDU does not granger cause LEX	4.442^**^	0.028
LEX does not granger cause EDU	1.783	0.198
HE does not granger cause LEX	3.305^***^	0.061
LEX does not granger cause HE	1.159	0.337
INF does not granger cause LEX	5.867^**^	0.012
LEX does not granger cause INF	1.727	0.208
GDP does not granger cause LEX	4.295^**^	0.031
LEX does not granger cause GDP	0.942	0.409
HE does not granger cause CO2E	6.314^*^	0.009
CO2E does not granger cause HE	5.025^**^	0.019
INF does not granger cause CO2E	0.732	0.496
CO2E does not granger cause INF	6.038^**^	0.010
GDP does not granger cause CO2E	1.701	0.212
CO2E does not granger cause GDP	3.765^**^	0.044

*, ** & *** indicates the significance level at 1, 5% & 10%.

## Conclusion and policy recommendation

This study empirically investigated the impact of air pollution on LEX in China from 2000 to 2023, focusing on the role of education and health expenditure using the ARDL approach to co-integration analysis and FMOLS and DOLS estimators for the Robustness analysis. The results revealed that education, health expenditure, and GDP positively affect LEX, while inflation and CO2E negatively influence LEX.

Based on these findings, the study offers several policy recommendations to improve health and social well-being, ultimately increasing LEX in China. First, more resources should be allocated to improve access to quality education, particularly in rural and underserved areas. Second, ensuring all citizens have access to education can enhance health literacy and promote healthier lifestyles, ultimately leading to longer life expectancy. Third, comprehensive health education should be incorporated into school curricula to teach students about healthy habits, disease prevention, and the importance of maintaining a balanced lifestyle. Fourth, allocate a more significant portion of the national budget to public health services to ensure universal access to healthcare. This will reduce health inequalities and improve LEX by providing timely medical care and disease prevention services. Six, invest in primary healthcare infrastructure to ensure all citizens can access basic healthcare services, especially in rural and underserved areas. Seven, strengthening primary care will prevent the onset of diseases and promote early intervention, which is essential for improving life expectancy. Eight, Allocate funds for preventive healthcare programs such as vaccination campaigns, health screenings, and public health education. These initiatives can reduce the burden of chronic and infectious diseases, leading to longer life expectancy. Nine, enforce stricter regulations on industrial emissions and vehicle pollutants to reduce CO2 emissions. This would help mitigate air pollution, a major contributor to respiratory and cardiovascular diseases, ultimately improving life expectancy. Ten: Invest in and incentivize the transition to renewable energy sources such as solar, wind, and hydropower. Eleven, reducing dependence on fossil fuels will lower CO2 emissions and help mitigate the adverse health effects associated with air pollution, contributing to longer LEX. Twelve, Promote the creation of more urban green spaces and trees to absorb CO2 emissions and improve air quality in urban areas. Green spaces also provide a healthier environment for physical activity, which can reduce the risk of chronic diseases and boost LEX.

Thirteen, enforce stricter emissions standards and promote clean energy adoption to reduce CO2 levels. Improve air quality monitoring and compliance to mitigate health risks. Fourteen, expand rural healthcare infrastructure and incentivize medical professionals to work in underserved regions. Subsidize essential services to ensure affordability for low-income populations. Fifteen, implement green taxation and incentives to balance industrial growth with environmental conservation and invest in healthcare and sustainable infrastructure to enhance long-term well-being.

This study has several limitations, first, the analysis is limited to the Chinese economy, which may restrict the generalizability of the results to other economic contexts. Future studies should consider expanding the scope to include developed, emerging, and developing nations, enabling a more comprehensive understanding of the factors influencing LEX across different economic and social structures. A cross-country comparison would provide valuable insights into regional variations and policy effectiveness. Second, the study utilizes a limited set of variables, focusing primarily on environmental and economic factors while omitting other critical determinants of LEX. Future research could incorporate a broader range of macroeconomic, demographic, social, and health-related variables, such as healthcare access, lifestyle factors, environmental conditions, and technological advancements in medicine. Third, methodological limitations exist, as the study does not account for asymmetric effects, Quantile regression, or structural break analysis. Asymmetric analysis could help identify whether the impact of economic factors on LEX differs during periods of economic growth versus downturns. Future research should integrate these advanced econometric techniques to obtain deeper insights into the dynamics of life expectancy determinants. Fourth, this study used CO2 emissions as a proxy for air pollution, though there are many other relevant indicators, such as PM2.5, NO₂, and SO₂. Future research should incorporate these additional measures to provide a more comprehensive analysis of air pollution. Finally, the data utilized in this study spans the period from 2000 to 2023, which may not fully capture long-term trends and cyclical patterns. Extending the time frame and incorporating historical data could improve the robustness of the findings and allow for better trend analysis.

## Data Availability

The raw data supporting the conclusions of this article will be made available by the authors, without undue reservation.
