# Supplementary material for: Air pollution and life expectancy: the role of education and health expenditure in China
Source: Front Public Health. 2025 May 7;13:1553039. doi: 10.3389/fpubh.2025.1553039 (PMC12092418; doi:10.3389/fpubh.2025.1553039)
Supplement: Supplementary file 1 [file Supplementary_file_1.docx]

Figure S1: Trends of life expectancy at birth rate in China.

Source: WDI

Figure S2: Normality test
